# A multiway analysis for identifying high integrity bovine BACs

**DOI:** 10.1186/1471-2164-10-46

**Published:** 2009-01-23

**Authors:** Abhirami Ratnakumar, Wesley Barris, Sean McWilliam, Rudiger Brauning, John C McEwan, Warren M Snelling, Brian P Dalrymple

**Affiliations:** 1 CSIRO Livestock Industries, 306 Carmody Road, St. Lucia, QLD 4067, Australia; 2 AgResearch, Invermay Agricultural Centre, PB 50034, Mosgiel, New Zealand; 3 USDA-ARS-NPA Roman L. Hruska U.S. Meat Animal Res, P.O. BOX 166, Clay Center, NE 6893, USA

## Abstract

**Background:**

In large genomics projects involving many different types of analyses of bacterial artificial chromosomes (BACs), such as fingerprinting, end sequencing (BES) and full BAC sequencing there are many opportunities for the identities of BACs to become confused. However, by comparing the results from the different analyses, inconsistencies can be identified and a set of high integrity BACs preferred for future research can be defined.

**Results:**

The location of each bovine BAC in the BAC fingerprint-based genome map and in the genome assembly were compared based on the reported BESs, and for a smaller number of BACs the full sequence. BACs with consistent positions in all three datasets, or if the full sequence was not available, for both the fingerprint map and BES-based alignments, were deemed to be correctly positioned. BACs with consistent BES-based and fingerprint-based locations, but with conflicting locations based on the fully sequenced BAC, appeared to have been misidentified during sequencing, and included a number of apparently swapped BACs. Inconsistencies between BES-based and fingerprint map positions identified thirty one plates from the CHORI-240 library that appear to have suffered substantial systematic problems during the end-sequencing of the BACs. No systematic problems were identified in the fingerprinting of the BACs. Analysis of BACs overlapping in the assembly identified a small overrepresentation of clones with substantial overlap in the library and a substantial enrichment of highly overlapping BACs on the same plate in the CHORI-240 library. More than half of these BACs appear to have been present as duplicates on the original BAC-library plates and thus should be avoided in subsequent projects.

**Conclusion:**

Our analysis shows that ~95% of the bovine CHORI-240 library clones with both a BAC fingerprint and two BESs mapping to the genome in the expected orientations (~27% of all BACs) have consistent locations in the BAC fingerprint map and the genome assembly. We have developed a broadly applicable methodology for checking the integrity of BAC-based datasets even where only incomplete and partially assembled genomic sequence is available.

## Background

BAC libraries are a key component of many large genomics projects. They are used in the construction of maps of regions of genomes see [1-5] for examples for the bovine genome, in the construction of maps of complete genomes [6-11], to provide a framework for the sequencing of genomes [12,13], and in comparative genomic hybridisation to study genome rearrangements [14,15]. Many projects undertake fingerprint and BES analyses to construct physical maps of the target genome; this information can also be used to identify a tiling path of BACs to be sequenced as part of a genome sequencing strategy. To enable a range of different analyses to be undertaken by different groups, several copies of the BAC library may be created or subsets re-arrayed with a number of different organisations undertaking various parts of the fingerprinting, BAC end-sequencing and full BAC sequencing, thereby potentially increasing the chances of BAC assignment errors.

**Figure 1:**
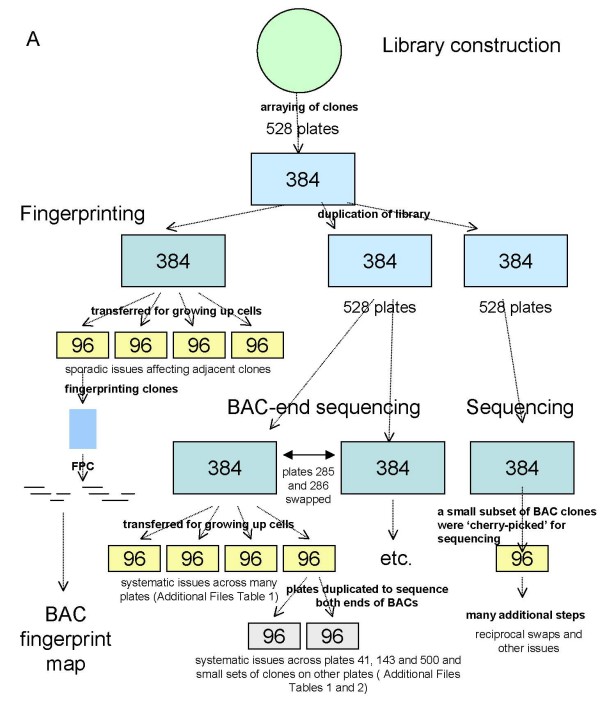
**Flow of BAC clones and DNA samples for the bovine BAC fingerprinting, BAC end sequencing and genome sequencing projects**. Results of the analyses undertaken in this publication are summarised. Numbers inside the boxes indicate the number of wells in the plates.

The route taken by the bovine genome project is defined as follows (Fig. 1). The CHORI library, CHORI-240 [16], was one of the major libraries used for the genome sequencing project [9]. It was fingerprinted at the British Columbia Cancer Agency Genome Sciences Centre and contigs were constructed from BACs with overlapping fingerprints [9]. These BAC contigs have been mapped to the bovine radiation hybrid [17] and composite maps [9], using markers assigned to BACs, largely by using BESs to create PCR probes. Smaller numbers of BACs from two other libraries, RPCI-42 [18] and TAMBT [19] were also included in the BAC fingerprint based map. A smaller set of CHORI-240 BACs have been included in a second BAC-fingerprint map [20]. Most plates in the CHORI-240 library were BAC end-sequenced with smaller numbers from the other libraries shared across a number of different laboratories [21]. Skim sequencing, typically 1.5× coverage, of approximately 10% of bovine BACs was undertaken as part of the bovine genome sequence at Baylor College of Medicine (BCM) [22].

Throughout the course of the bovine genome project the CHORI-240 library was replicated a number of times and different methods were used by several research groups at varying times on independent equipment. As part of the processing in these laboratories, clones were re-arrayed several times from 384 to 96 well plates for growth of cells prior to preparation of DNA and further split onto two 96 well plates for sequencing of the two ends of each BAC clone [21]. Despite this process being a frequent event there have been relatively few studies of the impact of these processes on the integrity of BAC assignments within large genomics projects. In an early assessment of the Human Genome Project, analyses of the association between clone name and sequence for the human BESs found a match rate for some sets of BACs of only 30% [23]. In a specific test of integrity 91% of clones contained the same BESs when determined at two different centres [23]. More recently during the construction of a set of BAC clones spanning the human genome approximately 7% of clones reanalysed did not generate the same fingerprint as generated in the original fingerprinting of the clones [24]. In a study using the mouse genome BAC-libraries, a consistency rate of 95% for repeat sequencing of BAC ends was observed [25]. The authors proposed that the high levels of automation in the processing pipelines should further increase the integrity of the datasets being generated [25]. Similar analyses of EST datasets also indicate a range of tracking error rates, almost 38% in a sample of the IMAGE cDNA clones [26], 11.1% in a set of bovine cDNAs [27] and ~7.6% in a set of honey bee cDNA clones [28]. In contrast, lane tracking errors during sequencing appear to be generally low, around 0.5% in a survey of a number of EST libraries [29].

A number of genome projects have used fingerprint maps, BESs and genome sequence data to identify sets of reliable BAC clones spanning the genome [24] or to build a BAC-based map of the genome [30]. In these projects the consistency between the BAC fingerprint and BES based positions on the genome was used to include or exclude BACs from either the set of BACs or the map. In the set of 73,305 paired end sequenced BACs positioned on the rat genome assembly 2% were assigned to different chromosomes by their fingerprint and BESs [30]. However, the source of the discrepancy, incorrect BAC fingerprint, or incorrect BES(s) was not reported.

**Figure 2:**
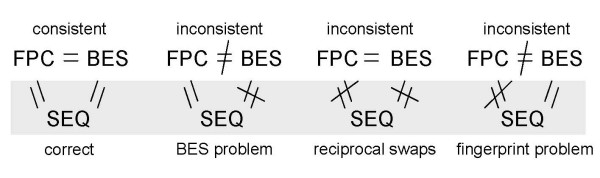
**Diagrammatic representation of the multi-way analysis of the BAC fingerprint map, BAC-end sequences and BAC sequences**.

The availability of the draft Btau3.1, and more recently the Btau4.0, assemblies of the bovine genome, which include sequence data derived from skim sequencing of a large number of BACs from the CHORI-240 library, provides an opportunity to determine the integrity of the BAC fingerprinting, BAC end sequencing and full BAC sequencing. In addition, it should also be possible to identify the most likely procedure during which a problem occurred. All three sets of data are incomplete, the Btau3.1 genome assembly is only a draft assembly, not all BACs have been fingerprinted and of those that have, not all have been included in contigs. Finally many BACs have only one or even no BES reads available (Table 1). By comparing the positions of the same BACs in the genome assembly, using the BESs and other non-BES data from the same BAC, and in the BAC fingerprint based genome, BACs can be divided into groups on the basis of consistency between the various combinations of the three datasets (Fig. 2). The challenge is to develop a methodology that enables the identification of problems and to make predictions about BACs for which we only have incomplete information. The high level of automation in the processing of the samples and the use of multi-channel pipettes etc. may help us do this since we might expect patterns of problematic BACs to be identifiable and thereby allowing us to make predictions based on patterns. Sporadic problems will be harder to detect and impossible to predict at the level of individual BACs.

**Table 1:** Numbers of fingerprinted and end sequenced BACs

	Fingerprinted^1^	BES
CHORI-240 library	202,752	
Fingerprinted	200,064	
Successful fingerprints	170,644	
Included in BAC fingerprint map	169,283	
In contigs in BAC fingerprint map	159,010^2^	
BAC end attempted		195,456^3^
at least 1 BAC end sequence		148,214
at least 2 BAC end sequences		121,314
At least one BAC end sequence and fingerprint	139,304	

^1^Numbers are from Snelling et al., 2007 [9]

^2^No clones from CHORI-240 library plates 205, 206 and 517 were included in the BAC fingerprint analysis. Less than half the average of 301 BACs per plate were included in the map from plates 516, 322 and 520 and 250 BACs or less were included from plates 310, 312, 324, 350 and 439.

^3^BACs on plates 479–497 were not end-sequenced

Here we undertake the three way comparison of BAC-based genomic datasets using the International Bovine BAC Mapping and Genome Sequencing Consortia datasets.

## Results

### Apparent reciprocal swaps in fully and partially sequenced BACs

The BAC end sequences from the bovine CHORI-240 BAC library were mapped to the draft bovine genome assembly Btau3.1 and the BACs were classified into those with two end sequences that map in the expected orientation and distance (tail-to-tail), those with two ends that map in conflicting orientations and/or distance, those with two ends located on different chromosomes (breaks) and those with only one end mapped (unpaired) as previously described [10] (Table 2). Available partial and complete sequences of the BACs were also mapped to the Btau3.1 genome assembly and BESs were mapped directly to the same set of partial and complete BAC sequences.

**Table 2:** Mapping bovine CHORI-240 BAC-end sequences to the bovine genome

BAC end sequences	Total	%	correct position	consistent position	correct and consistent position	inconsistent positions	% inconsistent
expected orientations and within size limit (tail-to-tail)	55,572	40%	3,826	46,295	50,121	2,932	5.3%
conflicting orientations and/or outside size limit same chromosome	8,666	6.2%	340	6,757	7,097	938	11%
two different chromosomes (breaks)	9,443^1^	6.8%	316	6,904	7,220	1,803	19%
only one BAC end placed on genome (unpaired)	64,996	47%	3,861	44,171	48,032	9,404	14%

Total	138,677	100%	8,343	104,127	112,470	15,077	11%

^1^does not include BACs with both end sequences mapped to the unknown chromosome

**Figure 3:**
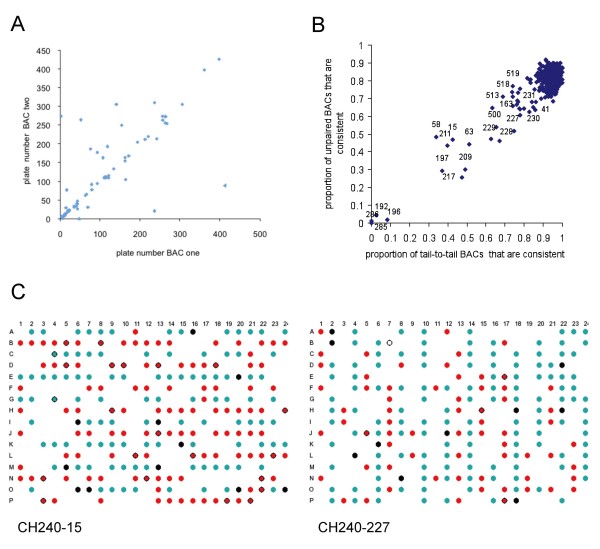
**Identification of plates with significant numbers of putative inconsistent BAC clones**. **A**. Plot of plate numbers of the BACs in putative reciprocal pairs plotted against one another. **B**. Plot of the proportion of consistent BACs in the unpaired group vs. the proportion of consistent BACs in the tail-to-tail group on a plate by plate basis. Numbers indicate plates with large numbers of inconsistent BACs. Plates without any tail-to-tail BACs were excluded from the plot. **C**. Diagrammatic representations of 384 well plates, CH240-15 and CH240-227. Correct BACs are indicated by black circles, consistent BACs are indicated by blue circles, putative non-consistent BACs are indicated by red circles and inconsistent BACs that have been sequenced are indicated by black rings around the red circle.

The sequenced BACs for which at least one BES was mapped to the bovine genome assembly were then divided into two groups: correct (sequence derived from the BAC maps to the vicinity of the corresponding independently determined BES(s)) and potentially incorrect. It was immediately clear that there were a substantial number of pairs of BACs with the BESs of BAC "A" mapped to genomic sequence flanking BAC"B" internal sequences and BAC "B" BESs mapped to genomic sequence flanking BAC "A" internal sequences. In some cases the BESs were also able to be directly aligned to the sequence of the putatively swapped BAC. However, due to the partial nature of many of the BAC sequences, the absence of this additional relationship was not diagnostic for the absence of a swap. Plotting the plate numbers of the pairs of putatively reciprocally swapped BACs (Fig. 3A) showed a very strong relationship between the plate numbers of the two BACs.

We then examined the possibility that systematic errors may have occurred at some point during the sequencing of the BACs. The BACs sequenced at BCM have been allocated internal sequencing project codes, which are included in the GenBank entries for the BAC sequences. A number of examples of pairs and up to six potentially swapped BACs with consecutive, or close to adjacent, BCM sequencing project codes were identified, suggestive of swapping of BACs during sequencing. Detailed examination of one of the groups of apparently swapped BACs identified an inverted relationship between the apparently swapped BACs and the BCM project codes: i.e. CHORI-240 BAC 66L13 (BCM project code FDVO) appeared to be swapped with 66O17 (FDVV), 66L18 (FDVP) appeared to be swapped with 66O12 (FDVU) and 66L21 (FDVR) appeared to be swapped with 66M24 (FDVS). However, many of the potentially incorrect BACs did not appear to have a simple reciprocal relationship with a single other BAC, for example many of these BACs apparently contained DNA derived from two different BACs (data not shown). These relationships are potentially very complex and as many of the BAC sequences are still only draft sequences we have not attempted to resolve all of the discrepancies in this dataset.

### Identification of inconsistencies between the BAC end sequence and the BAC fingerprint determined positions

Since only a small number of BACs have been sequenced a much broader analysis was required to identify the range of potential issues with the integrity of the unsequenced BACs. To undertake this analysis all of the BES mapped positions on the bovine genome were converted to approximate locations on the BAC fingerprint based map as described in the methods. BACs with equivalent positions in both maps were called consistent, and BACs with apparently different positions in the two maps were called potentially inconsistent. There are many ways that BACs could be in the potentially inconsistent set, for example; errors in our classification pipeline, errors in the fingerprinting or BAC end sequencing, errors in the assembly of the BAC fingerprint map, or in the positioning of the BES(s) on the bovine genome assembly. The latter is clearly likely to be a significant problem for BACs with only one end positioned (unpaired), or with their ends positioned on two different chromosomes (breaks). Indeed, based on just one end sequence (unpaired), or testing both end sequences (breaks) BACs in both of these groups contained a significantly higher percentage of inconsistent BACs, 14–19%, than the rate of 5.3% in the BACs that map with the expected organisation. However this still suggests that the locations of most unpaired BESs in the bovine genome assembly are indeed correct and for the majority of the BACs with BESs mapped to different chromosomes one end is correct and that the location of the BAC in the fingerprint map can be used to identify the correct position in the genome assembly.

However, inconsistent BACs may have arisen by either sporadic or systematic problems. In order to identify systematic issues, the distribution of the proportions of consistent BACs for the set of tail-to-tail BACs and the set of unpaired BACs were plotted against each other (Fig. 3B). Overall the two sets of values are highly correlated, for the majority of the plates almost all of the tail-to-tail BAC clones were consistent. However, there are clearly a number of plates which contain much higher numbers of potentially inconsistent BACs, for example almost all BACs on plates 285 and 286 appeared to be potentially inconsistent (Fig. 3B). The plates lying outside the bulk of the data points were examined in more detail. To facilitate the analysis, and to identify if systematic errors were involved, the locations of the consistent and potentially inconsistent BACs were plotted on graphics of the plates, examples are shown in Fig. 3C. The results of the complete analyses are shown in the Additional Files 1 (Table 1). In all cases errors during the BAC-end sequencing appear to have been the major cause for the conflicts between the BAC fingerprint map and BES positions. This was determined based on the category of the sequenced BACs which lay in a row or column of potentially inconsistent BACs on a plate with systematic errors. If these BACs were predominantly in the correct group (i.e. BESs matching full sequence) then it is likely that the discrepancy between the BES and the fingerprint was due to errors in the fingerprinting, conversely if none of these BACs were in the "correct" group then it is most likely that the errors occurred during the BAC-end sequencing.

In summary, for this set of BACs both end sequences were derived from a different BAC from the one intended to be end sequenced, but each end of each BAC was sequenced only once.

During the calculations of the ratios described above we observed that nine plates of BACs did not contain any BACs in the tail-to-tail group. On seven of these plates: 10, 345, 477, 478, 520, 521 and 522 only sequences from one end had been deposited in GenBank and therefore BACs could not be in the tail-to-tail set. However, BESs from both ends of the BACs on plates 446 and 447 had been deposited in GenBank, further analysis (comparing the locations of the BAC ends and the BACs in the BAC fingerprint based map) suggested that the TARBAC13P2 primed sequence reads have been swapped between these two plates (Additional Files 1: Table 1).

In summary, one BES from the pair of BESs that correspond to each BAC on plate 446 was swapped with one BES from each pair of BESs for each equivalently positioned BAC on plate 447.

### Analysis of BACs derived from the same CHORI-240 library plate that have overlapping BESs on the bovine assembly

In the above analysis we assumed that both BESs for potentially inconsistent BACs were "unique" in the library, barring the occasional use of exactly the same restriction enzyme site at the vector-clone junction. However, it is possible that single sets of BESs could have been determined twice and assigned to two different sets of BACs. The way in which the analysis was undertaken, in particular allowing either end of a BAC with end sequences mapping to two different chromosomes to be consistent with the BAC fingerprint map location, would have obscured these cases (Fig. 2). In order to address this issue the set of locations of CHORI-240 BESs mapped to the bovine assembly were scanned for overlapping BESs. 17,577 pairs of BACs with at least one BES overlapping BESs from one or two other BACs were observed, in 2,628 of these BAC pairs both BACs were derived from the same CHORI-240 plate, about 70 times more than expected from a random distribution of overlapping BESs. 3,367 BACs from the 2,628 BAC pairs were in the consistent set, and 1,218 of the BAC pairs from the same plate with at least one overlapping BES also have overlapping fingerprints.

**Figure 4:**
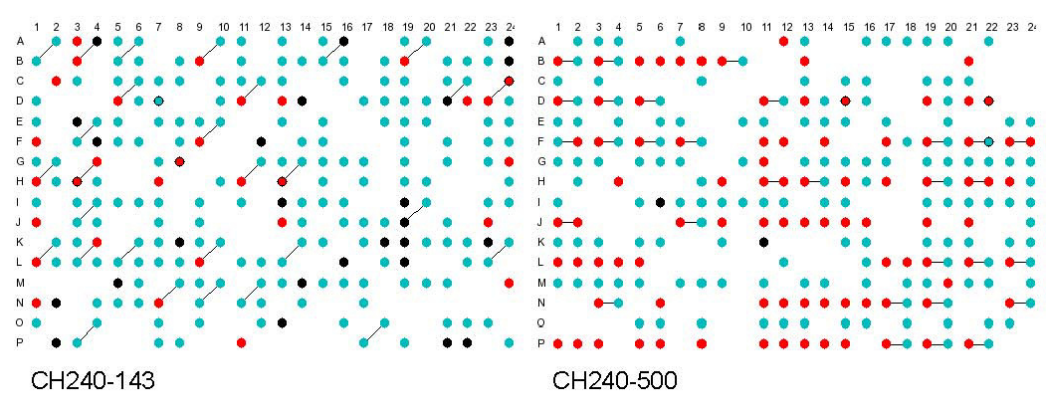
**Analysis of BACs with overlapping BAC-end sequences**. Diagrammatic representations of 384 well plates, CH240-143 and CH240-500. BAC colour coding is the same as for Figure 4. Black lines join wells containing BACs with one or more overlapping BAC end-sequences.

Two types of systematic relationships were identified; plates where at least some BACs in a consistent pattern had both ends overlapping with another BAC from the same plate, where one BAC was consistent and the other was not, and plates where there was a pattern of overlapping BESs that included consistent BACs from the breaks group and inconsistent BACs from the unpaired group. In the former case both ends of a set of BACs appear to have been sequenced twice (for example, plates CH240-15 and CH240-227, Fig. 3C), in the latter case one end of a group of BACs appears to have been sequenced twice generating BACs with end sequences probably derived from two different BACs (for example, plates CH240-143 and 500, Fig. 4). These mis-identities appear to have occurred at a number of levels, complete 96 well plates, individual rows and duplication of half a row (Additional Files 1: Tables 1 and 2).

In summary, in this set of BACs one end sequence was derived from a different BAC from the one intended to be end sequenced and one end of many of the BACs was sequenced twice or the data was erroneously deposited in the database twice.

### Analysis of BACs derived from the same CHORI-240 library plate that overlap in the BAC fingerprint map and in the genome assembly

**Figure 5:**
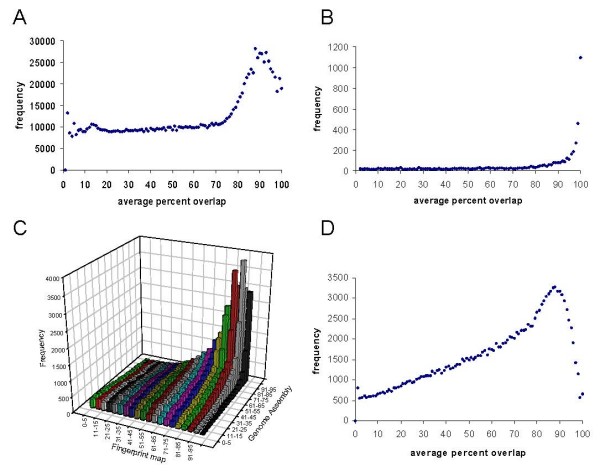
**Analysis of BACs with overlaps**. **A**. Distribution of average overlap lengths of all overlapping BACs in the BAC fingerprint map, excluding BACs from the same plate in the CHORI-240 library. **B**. Distribution of average overlap lengths of all overlapping BACs from the same plate in the CHORI-240 library. **C**. Distribution of average overlaps from BAC fingerprints v average overlap in the genome assembly. **D **Distribution of average overlap lengths in the genome assembly of all BACs with overlaps in both the BAC fingerprint map and the genome assembly.

The large number of BACs with overlapping BESs prompted us to undertake a more detailed analysis of the overlapping BACs in the BAC fingerprint based map. The sizes of the overlaps between the BACs included in the map were calculated. The dataset was then divided into two sets; overlapping BACs derived from different CHORI-240 library plates (Fig. 5A), and BACs derived from the same plate (Fig. 5B). The number of overlapping CHORI-240 BACs derived from the same plate was 9,436, almost double the expected numbers of 4,768 based on the average of ~15.74 overlaps per BAC and of 4,802 calculated by randomisation of the actual dataset. Across the whole library an excess of BACs is clearly visible for overlaps greater than 75%, reaching a peak at almost three fold greater than the average at 90% overlap (Fig. 5A). However, overlapping BACs within plates show an even greater excess of highly overlapping clones, again diverging from the background at around 75% overlap, but with a maximum of ~50 fold excess at 100% overlap.

Although the BAC fingerprint based map of the genome assembly displays details for a very large number of BAC clones the lengths of clones and the extent of the overlaps are not highly accurate. This can be illustrated by a comparison of the overlaps of pairs of clones based on the mapping of the BAC ends to the genome assembly (tail-to-tail clones only) and from the BAC fingerprint based map (Fig. 5C). Because the analysis uses only BAC clones for which both sets of data are available a much reduced number of BAC clones are included. Overall there is a trend with a correlation coefficient of ~0.51, but clearly the BAC-fingerprint overlap information is only indicative of the true extent of the overlap. The distribution of the BAC overlaps based on the genome assembly also shows a pronounced peak at 85–88% average overlap (Fig. 5D). Of the overlapping BACs derived from the same plate 56% had overlaps of 99% or more based on the positions of their BESs in the genome assembly.

**Figure 6:**
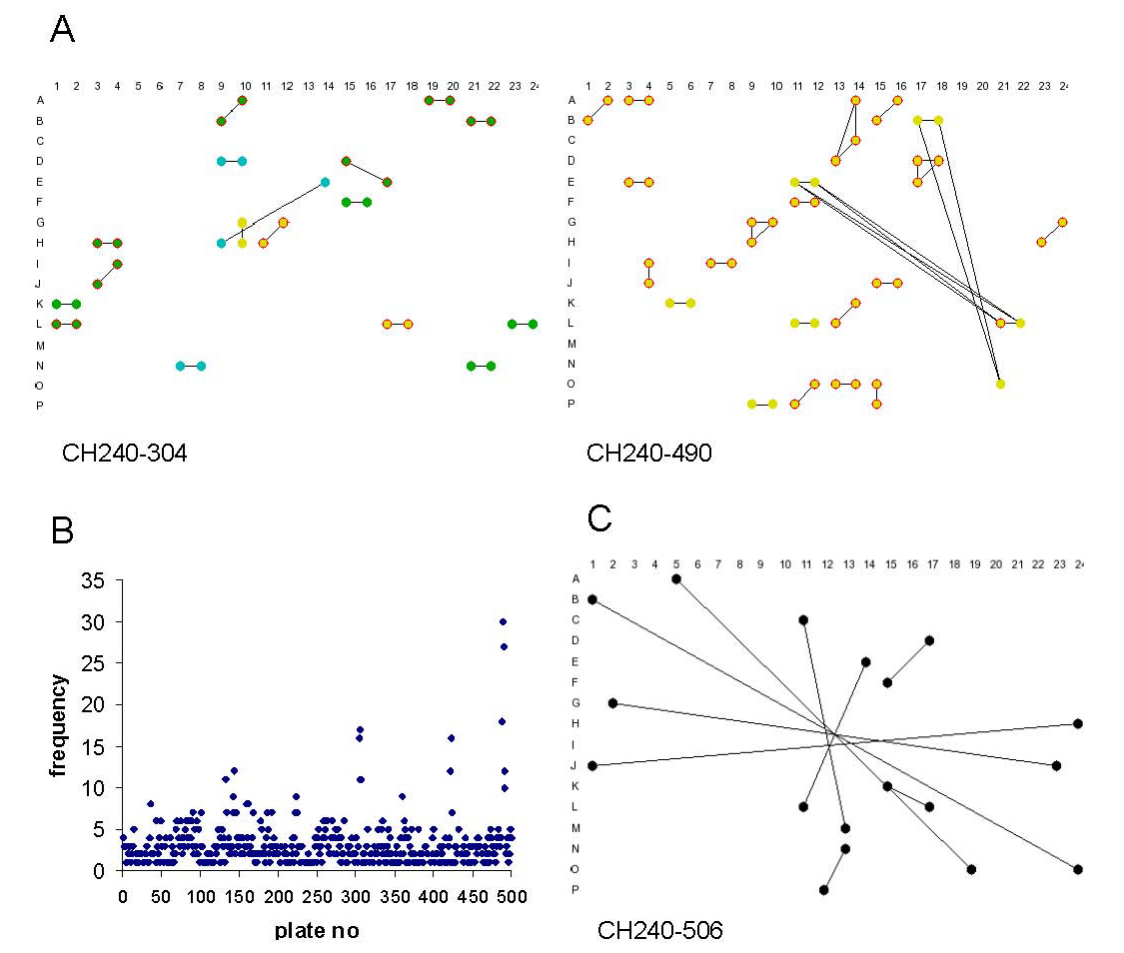
**A. Diagrammatic representations of two plates with high rates of plate wide systematic overlapping BAC fingerprints, but not high rates of putative inconsistent BACs**. BAC colour coding is as follows; fingerprint overlap and BAC end sequence overlap – green, fingerprint overlap and BAC end sequence known, but no overlap – cyan, fingerprint overlap and BAC end sequence overlap not known – yellow. Red outline fingerprint overlap greater than 90%. Black lines join wells containing BACs with overlapping fingerprints. **B**. Plot of the frequency of BACs from the same plate that overlap in the fingerprint map and are located on the same row and 1 or 2 columns apart, the same column and 1 or 2 rows apart or at a diagonal 1 or 2 wells part. **C**. Example of a plate exhibiting a star-like relationship between wells with overlapping BAC-end sequences.

Since just over half of the BACs in an overlapping pair were also correct, or consistent, the majority of the within plate, highly overlapping, BACs appear to be stable and therefore likely to have arisen during the original construction of the library. To identify the patterns, if any, for these processes we generated graphics for a number of plates with large numbers of BACs with overlapping FPC locations (Fig. 6A). In general these plates, such as CH240-304 and CH240-490 contained mainly pairs of BACs with overlapping fingerprints where the BACs were located on the same row in adjacent columns, or diagonally one row and one column apart. In addition, all plates contained pairs of BACs with an apparently random relationship.

To further investigate the apparent systematic relationship between pairs of overlapping clones observed on some plates based on the observation above and elsewhere in the analyses described here, we plotted the frequency of overlapping BACs in the same row one or two columns apart, in the same column, one or two rows apart and the corresponding diagonal relationship (Fig. 6B). This identified a number of other plates with apparently systematic relationships between overlapping clones. The nature of the patterns suggests that these relationships were generated during transfer between 384 and 96 plates or vice versa. However, in the majority of cases the relationships appeared to be random suggesting that in the cases of substantial overlap these were more likely to be due to multiple clone picks rather than contamination per se.

Another interesting, but very infrequent pattern, that was observed by linking BACs with overlapping BAC-end sequences on the plate was star-like (Fig. 6C). Although only a small proportion of the BACs on plates were affected, both end sequences of one of the BACs involved overlapped with the equivalent end sequences of the other BAC. In almost all cases BAC fingerprint data was not available for both BACs involved, so the point at which the apparent contamination occurred cannot be determined. The nature of the relationships appears to indicate a 180 degree rotation of the original plate, or copies of the plate, or perhaps a lid, that ultimately affected the BAC-end sequences from only a small number of the wells.

In summary, the majority of overlapping BACs located on the same plate in the BAC library are likely to be two examples of exactly the same BAC clone, perhaps arising from replication of clones prior to plating or picking of the same clone twice during the transfer to the clones.

## Discussion

Since it will never be possible to identify all of the issues, a list of all BACs with questionable identities will never be complete, however, using the approach above we can define a set of bovine BACs with a high probability of being reliably recovered from copies of the CHORI-240 library using a few rules;

• Avoid using BACs that do not have both a fingerprint and at least one BES available (where both methods have been used) – check that the fingerprint and genome positions are equivalent

• Do not use more than one of a set of BACs with substantial overlaps in the BAC fingerprint based map, in the genome assembly, or BESs, where the BACs are derived from the same library plate, as it is likely that the BACs are identical

• Do not use BACs from library plates with systematic issues

• Never use BACs with conflicts between the position in the fingerprint-based map and genome assemblies, and/or DNA sequence if available

These rules are based on the assumption that at least the fingerprinting and end sequencing of the BACs has been undertaken directly from the original library plates, or replicates that maintain the original relationships.

• The most preferable BACs are those where the DNA sequence and BES-based positions on the genome assembly and the fingerprint-based map positions are all consistent

• If no sequenced BACs are available for a region of interest only use BACs where the BES (tail-to-tail) and fingerprint positions are available and are consistent.

During fingerprint map construction BAC clones with extremes of insert size and number of restriction fragments were excluded from the analysis to screen out potentially mixed wells [21], thus clones incorporated into the map are unlikely to be heavily contaminated. Therefore BACs excluded from the final fingerprint map should not be used for further characterisation, even when they have BES data.

It has been reported that the draft Btau3.1 assembly of the bovine genome has a number of problems and could be substantially improved [21,31]. This leads to the question; have issues with the identities of the BACs contributed to errors in the assembly and/or to the perception of the extent of the potential errors in the assembly? Of the 12,671 CHORI-240 library BAC clones with sequence data deposited in GenBank and used in this study 413 were from wells identified as likely to contain BACs with one or more end-sequence that was not derived from the same BAC as the fingerprint. Since these appear to have resulted from systematic errors during the BAC-end sequencing the identities of the BACs sequenced during the genome sequencing project should be correct. Since the assembly of the genome is based on the sequence and not the identity of the BACs per se these will not have impacted on the assembly of the genome sequence. Only sixteen BACs with one apparently correct and one apparently incorrect end sequence were included in the genome sequencing. A much larger number of such BACs, contributing incorrect mate pair information, were included in the set of BAC end sequences in the initial pool of sequences used in the assembly. Since multiple consistent mate pair links are required to link contigs within scaffolds during genome assembly it is unlikely that this relatively small set will have significantly contributed to assembly problems, rather just added to the background noise. Due to the relatively small numbers of BACs involved it is also likely that these had little impact on the comparison of the genome sequence and BAC fingerprint based map [9]. However, our analysis suggests that only a single BAC mate pair link should be allowed from any one plate in the BAC library to avoid the high rate of apparently the same clone on the same plate contributing to the generation of incorrect links.

A detailed analysis of the large number of consistent non-tail-to-tail BACs has demonstrated that most of these resulted from the limitations of the draft assembly of the bovine genome used in the analysis, Btau3.1. During the course of this work a revised assembly of the bovine genome, Btau4.0, was released, which has resolved many of these limitations. However, our analysis methodology was designed to be robust, allowing for assembly errors by using non-tail-to-tail BACs, requiring the position of only one BES to be consistent with other data sets and allowing 500 kb windows for matches. Preliminary investigations indicate that the new bovine assembly has had no impact on the identification of the problematic BES plates. Small numbers of BACs currently identified as incorrect may now be classified correct using Btau4.0, however BACs previously identified as correct and consistent remain as correct or consistent.

There are many places where errors can arise in large genome sequencing projects. However, as we have outlined it is also possible to identify problems and correct errors. Overall, the error rate for bovine BACs with both fingerprints and end sequences appears to be less than 5%, consistent with expectations [25]. By applying the rules described here the risk of inadvertently characterising an incorrect BAC clone can be reduced to virtually zero. The rules and methods described here are applicable to all such datasets and analyses.

## Methods

### Mapping BAC-end and full BAC sequences to the bovine genome assembly

The set of bovine BAC-end sequences downloaded from GenBank was filtered as described previously [21] and aligned to the bovine genome, build Btau3.1, using MegaBLAST with the following parameters: -F "m D" -U T -D 2 -m 8. The bovine genome sequence assemblies were obtained from the UCSC Genome Bioinformatics site [32,33]. The BESs were grouped into tail-to-tail, tail-to-head etc. as previously described [10]. The BAC sequences were aligned to the bovine genome using MegaBLAST with the parameters: -D 3 -W 32 -F m -U T -e 1e-100. A subset of BESs were also mapped to the available BAC sequences using MegaBLAST with parameters -D 3 -W 32 -F m -U T -e 1e-100.

### Identification of correct, incorrect, and reciprocally swapped sequenced BACs

The coordinates from the mapping of the BESs and BACs to the bovine genome were compared. Sequenced BACs with BAC ends that mapped within 250 kb either side of the region of the bovine genome containing the corresponding BAC sequence were called correct. Sequenced BACs with BAC ends that did not meet this criterion were called potentially incorrect, and the process below was used to determine whether they were consistent or potentially inconsistent. Reciprocal swaps were identified by comparing the locations of the BESs within 250 kb either side from the potentially incorrect BACs with the locations of the BAC sequences for the same set of potentially incorrect BACs. If at least 1BES of BAC A mapped to a location within 250 kb of the location of BAC B (other than itself), and at least 1 BES from BAC B mapped to within 250 kb of the position of BAC A, then BAC A and B are called potentially reciprocally swapped BACs.

### Identification of consistent and inconsistent BACs

The BAC fingerprint map data (version May 1, 2006) was downloaded from BCGSC [34]. Consistent BACs were identified by taking the BES positions for BACs that were potentially incorrect from the previous step and finding all sequenced BACs and BACs with BESs positioned tail-to-tail that overlap with either BES (with 250 kb leeway) of this potentially incorrect BACs set. Then the BAC fingerprint map positions of the BACs that were found to overlap with the starting BACs were determined. The fingerprint map location for cases where more than one BAC from the same fingerprint map contig was found to overlap with the potentially incorrect BAC was determined by taking the minimum start and maximum end coordinates for the set of BACs. The fingerprint map positions for these BACs that overlap with the potentially incorrect set were used to determine which other BACs overlapped with them in the fingerprint map. If any of the members of the final list of overlapping BACs in the fingerprint map were the same as the BAC from the initial potentially incorrect set the initial BAC was called consistent. However, if none of the overlapping BACs were the same as the initial BAC, the BES and fingerprint positions for the initial BAC are not consistent, we called these BACs putative non-consistent.

### Identification of BAC end sequence overlaps

The size and number of the overlaps between all of the CHORI-240 BESs mapped to the bovine genome were calculated using Perl scripts. Each of the positioned BES locations were compared to all of the other BES locations, cases where there was overlap between BESs from 2 different BACs were recorded. The results for BACs with overlapping BESs from the same plate were determined by comparing the plate number embedded in the BAC clone ids.

### Calculation of BAC fingerprint map and genome sequence overlaps

The number and the average percent of overlap between all pairs of the CHORI-240 BACs in the bovine BAC fingerprint map were calculated from the coordinates in the BAC contig file using Perl scripts. For each pair of overlapping BACs the average percent overlap was calculated from the individual percent overlaps calculated for each of the two BACs. The same scripts were used to calculate the set of average percent overlaps derived from the bovine genome assembly (Btau4.0). A filtered set of overlaps between BACs derived from the same plate in the BAC library was also generated by comparing the plate number embedded in the BAC clone ids. To calculate the expected frequency of overlapping BACs on the same plate the BAC-names in the full dataset were randomised independently 10 times and the background frequency of within plate overlaps calculated by comparing the plate number embedded in the BAC clone ids.

### Access to data

The high integrity BACs are displayed on the Btau4.0 genome browser along with the mapping of the bovine BAC-ends to the bovine genome assembly [35]. A list of high integrity BACs and the images of the BAC-end sequence overlaps and BAC fingerprint overlaps are also available from the livestock genomics website [36].

## Abbreviations

BAC: Bacterial Artificial Chromosome; BES(s): BAC end sequence(s); BCM: Baylor College of Medicine.

## Authors' contributions

AR carried out the majority of the analysis and helped to draft the manuscript. WB, SMcW and RB carried out some of the analyses, JMcE and WS and BPD conceived and designed the study and BPD coordinated the study and drafted the manuscript. All authors read and approved the final manuscript.

## Supplementary Material

Additional file 1 **Table 1.** Plates identified as containing BACs with plate-wide systematic identity integrity issues. Rows and/or columns containing putative inconsistent BACs are shown, along with whether the inconsistency is likely to have arisen during fingerprinting or end sequencing. The table also shows the number of BACs on the plate predicted to have systematic identity integrity issues, along with the number of BACs that have been fully sequenced.
**Table 2.** Plates identified as containing more localised sections of BACs with systematic identity integrity issues. Rows and/or columns containing the putative inconsistent BACs are shown, along with the affected wells, the number of observed affected clones and the number of BACs that have been fully sequenced. Click here for file
