# Supplementary material for: A multiway analysis for identifying high integrity bovine BACs
Source: BMC Genomics. 2009 Jan 23;10:46. doi: 10.1186/1471-2164-10-46 (PMC2660975; doi:10.1186/1471-2164-10-46)
Supplement: Additional file 1 — Table 1. Plates identified as containing BACs with plate-wide systematic identity integrity issues. Rows and/or columns containing putative inconsistent BACs are shown, along with whether the inconsistency is likely to have arisen during fingerprinting or end sequencing. The table also shows the number of BACs on the plate predicted to have systematic identity integrity issues, along with the number of BACs that have been fully sequenced. Table 2. Plates identified as containing more localised sections of BACs with systematic identity integrity issues. Rows and/or columns containing the putative inconsistent BACs are shown, along with the affected wells, the number of observed affected clones and the number of BACs that have been fully sequenced. [file 1471-2164-10-46-S1.doc]

**Table 1.** Plates identified as containing BACs with plate-wide systematic identity integrity issues.

| Plate | Putative inconsistent BACs | End sequencing or fingerprinting issue | Sequenced  BACs^1^ | Affected clones predicted |
| --- | --- | --- | --- | --- |
| 15 | BACs on alternate rows B,D,F,H,J,L,N,P | End-sequencing | 19 | 192 |
| 19 | BACs at the intersection of rows B,D,F,H,J,L,N.P and even numbered columns | End-sequencing - reads from these wells may be a repeat of the preceding odd numbered BACs in the same row | 4 | 96 |
| 41 | BACs at the intersection of rows B,D,F,H,J,L,N,P and odd numbered columns | End-sequencing – reads from these wells may be a repeat of the following even numbered BACs in the same row | 2 | 96 |
| 58 | BACs on alternate rows B,D,F,H,J,L,N,P | End-sequencing | 12 | 192 |
| 63 | BACs at the intersection between rows A,C,E,G,I,K,M,O and even numbered columns and at intersection between rows B,D,F,H,J,L,N P and odd numbered columns | End-sequencing | 11 | 192 |
| 143 | BACs at the intersection of rows B,D,F,H,J,L,N,P and odd numbered columns | End-sequencing – reads from these wells may be a repeat of the following even numbered BAC in the preceding row | 7 | 96 |
| 163 | BACs at the intersection between rows A,C,E,G,I,K,M,O and even numbered columns | End-sequencing | 6 | 96 |
| 187 | BACs at the intersection of rows B,D,F,H,J,L,N.P and even numbered columns | End-sequencing - reads from these wells may be a repeat of the preceding odd numbered BACs in the same row | 7 | 96 |
| 192 | All BACs except possibly at the intersection of rows A-H and columns 21 to 24 | End-sequencing | 14 | 384 |
| 195 | BACs at the intersection of rows B,D,F,H,J,L,N,P and even numbered columns, except possibly at the intersection of rows A-h and columns 13-24 | BAC-end sequencing? - several correct BACs in predicted inconsistent locations, or partial correction of end-sequencing errors? For example 195H18 and 195H20 appear to have the same BAC-end sequences as do a number of other BACs in a similar relationship | 6 | 96 |
| 196 | All BACs except possibly at the intersection of rows A-H and columns 21 to 24 | End-sequencing | 26 | 384 |
| 197 | BACs at the intersection of rows A, C, E, G, I, K, M and O and even numbered columns and at the intersection of all rows with odd numbered columns, except possibly at the intersection of rows A-H and columns 21 to 24 | End-sequencing | 23 | 288 |
| 209 | BACs at the intersection of rows B, D, F, H, J, L, N and P and odd numbered columns and at the intersection of all rows with even numbered columns, except possibly at the intersection of rows A-H and columns 21 to 24 | End-sequencing | 18 | 288 |
| 211 | BACs on alternate rows A,C,E,G,I,K,M,O except possibly at the intersection of rows A-H and columns 21 to 24 | End-sequencing | 8 |  |
| 217 | BACs at the intersection of rows A, C, E, G, I, K, M and O and even numbered columns and at the intersection of all rows with odd numbered columns, except possibly at the intersection of rows A-H and columns 21 to 24 | End-sequencing | 8 | 288 |
| 218 | BACs at the intersection of rows A,C,E,G,I.K,M,O and odd numbered columns except possibly at the intersection of rows A-H and columns 21 to 24 | End-sequencing | 2 | 96 |
| 219 | BACs at the intersection of rows A,C,E,G,I.K,M,O and odd numbered columns except possibly at the intersection of rows A-H and columns 21 to 24 | End-sequencing | 3 | 96 |
| 220 | BACs at the intersection of rows B,D,F,H,J,L,N.P and even numbered columns | End-sequencing - reads from these wells may be a repeat of the preceding odd numbered BACs in the same row | 1 | 96 |
| 227 | BACs in odd numbered columns except possibly at the intersection of rows A-H and columns 21 to 24 | End-sequencing | 5 | 192 |
| 228 | BACs at the intersection of rows A, C, E, G, I, K, M and O and even numbered columns and at the intersection of B, D, F, H, J, L, N and P and odd numbered columns, except possibly at the intersection of rows A-H and columns 21 to 24 | End-sequencing | 11 | 192 |
| 229 | BACs on alternate rows B,D,F,H,J,L,N,P, except possibly at the intersection of rows A-H and columns 21 to 24 | End-sequencing | 2 | 192 |
| 230 | BACs at the intersection between the rows B,D,F,H,J,L,P and odd-numbered columns, except possibly at the intersection of rows A-H and columns 21 to 24 | End-sequencing | 5 | 96 |
| 231 | BACs at the intersection between the rows B,D,F,H,J,L,P and even-numbered columns | End-sequencing | 1 | 96 |
| 285 | Swapped with plate 286^2^ | End-sequencing - but only two BACs sequenced | 2 | 384 |
| 286 | Swapped with plate 285^2^ | End-sequencing - but only three BACs sequenced | 3 | 384 |
| 446 | All BACs affected, but does not directly contribute to inconsistency | End-sequencing – reads with primer TARBAC13P2 may have been swapped with equivalent reads on plate 447 |  | 384 |
| 447 | All BACs affected, but does not directly contribute to inconsistency | End-sequencing – reads with primer TARBAC13P2 may have been swapped with equivalent reads on plate 446 |  | 384 |
| 498 | BACs at the intersection between rows A,C,E,G,I.K,M,O and even-numbered columns | End-sequencing – but few sequenced BACs | 1 | 96 |
| 499 | BACs at the intersection between rows A,C,E,G,I.K,M,O and even-numbered columns | End-sequencing – but few sequenced BACs | 1 | 96 |
| 500 | BACs at the intersection of rows B,D,F,H,J,L,P and odd numbered column | End-sequencing – reads from these wells may be duplicates of reads from the following even numbered column in the same row | 1 | 96 |
| 517 | BACs in rows A, C, E, G, I, K, M and O and BACs in rows B, D, F, H, J, L N and P one column to the right of the BAC in the preceding row | Both end sequences of the pairs BACs overlap |  | 384 |

^1^Sequenced BACs in rows and columns affected by the putative systematic identity integrity issues.

^2^PCR-based analysis of representative BACs from plates 285 and 286 is consistent with the plates being swapped.

Table 2. Plates identified as containing BACs with more localised systematic identity integrity issues.

| plate | systematic patterns | affected wells | sequenced | no. of affected clones observed |
| --- | --- | --- | --- | --- |
| 135 | BACs in row O, even columns | End sequencing – reads in wells in columns 2, 4, 6, 8,10 and 12 may be repeats of the reads in columns, 24, 22 ,20,18,16 and 14 | none | 6 |
| 155 | BACs in rows L and N, even columns | End sequencing – reads in the wells in row L may be a duplicate of the reads in the equivalent wells in row N. | 1 | 8 |
| 158 | 2 wells apart vertical | rows E and G, even numbered columns | 1 | 6 |
| 170 | BACs in rows J and L, odd columns | End sequencing – reads in the wells in row J may be a duplicate of the reads in the equivalent wells in row L. | 3 | 12 |
| 195 | 2 wells apart horizontal, 2 wells apart diagonal | columns 18-20 | 3 | 20 horizontal, 14 diagonal |
| 304 | 1 well apart horizontal, 1 well apart diagonal | various | 4 | 16 horizontal, 8 diagonal |
| 305 | 1 well apart diagonal, 1 well apart horizontal |  | none | 16 horizontal, 12 diagonal |
| 306 | 1 well apart horizontal, 1 well apart diagonal |  | 1 | 12 horizontal, 6 diagonal |
| 307 | 1 well apart horizontal, 1 well apart diagonal |  | 3 | 12 horizontal, 6 diagonal |
| 410 | 2 wells apart vertical | End sequencing - reads in the wells in the odd numbered columns in row J may be a duplicate of the reads in the equivalent wells in row L. | none | 18 |
| 413 | BACs in rows F and H and even numbered columns | End sequencing | none | 14 |
| 422 | 1 apart horizontal, 1 well apart diagonal |  | none | 16 horizontal, 6 diagonal |
| 423 | 1 apart horizontal, 1 well apart diagonal |  | none | 16 horizontal, 8 diagonal |
| 424 | 1 apart horizontal, 1 well apart diagonal |  | none | 10 horizontal, 8 diagonal |
| 501 | BACs in rows I and K and even numbered columns | End sequencing | none | 12 |
